# Honorable Mention

**Published:** 1890-12

**Authors:** 


### Honorable Mention.

Dr. W. G. Melotte, the well-known dentist of Ithaca, N. Y., gave interesting clinics before the Dental Section of the International Medical Congress, at Berlin, in which he illustrated his peculiar methods and specialties to the great interest of those who witnessed his methods. He also had the opportunity of presenting the same demonstrations before the British Dental Association at Exter, England. He also met many of the profession in Paris, where he was received with marked distinction.

Dr. Melotte is the inventor of a number of valuable appliances and methods employed in dentistry, all of which are well known in this country, and it is well that dentists in other countries should have the benefit of his inventions and devices.

